# Editorial: Glial cells: mere passive contributors to brain function?

**DOI:** 10.3389/fncel.2023.1360462

**Published:** 2024-01-30

**Authors:** Donaji Chi-Castañeda, Edna Suárez-Pozos, Arturo Ortega

**Affiliations:** ^1^Instituto de Investigaciones Cerebrales, Universidad Veracruzana, Xalapa, Veracruz, Mexico; ^2^Department of Anatomy and Neurobiology, Virginia Commonwealth University, Richmond, VA, United States; ^3^Laboratorio de Neurotoxicología, Departamento de Toxicología, Centro de Investigación y de Estudios Avanzados del Instituto Politécnico Nacional, Ciudad de México, Mexico

**Keywords:** astrocytes, central nervous system, microglia, neurodegenerative diseases, oligodendrocytes, therapeutic agents

Since first described in the 19th century, glial cells were believed to function as the non-functional glue that kept neurons together. Nowadays, we know that these non-neuronal cells are actively involved in a plethora of functions related to health and disease, including modulation of the nerve signal propagation rate, control of neurotransmitter traffic that results in synaptic modulation, and modulation and maintenance of nerve cells' ionic equilibrium. Moreover, glial cells function as scaffolds for neural development and recovery after neural injury.

Glial cells constitute 33%−66% of the brain mass and, in the mature central nervous system (CNS), can be classified into three types: astrocytes, oligodendrocytes, and microglia, each with specific functions. Although the general aspects of these cells are well described, the need to better characterize the function of specific populations under normal and pathological conditions remains. Advances in science and the development of newer and more powerful techniques have allowed us to discover more specific aspects and functions of the different types of glial cells.

The present Research Topic contains *state-of-the-art* reviews, original research, and perspectives that feature current advances and approaches to elucidate the role of glial cell in diverse physiological and pathological conditions.

Microglia are the main immune cells of the CNS. Accumulating evidence suggests that obesity, prediabetes, and diabetes predispose individuals to CNS complications through chronic low grade systemic inflammation, which extends to the brain and, coupled with a high-fat diet (HFD), causes an inflammatory phenotype in hippocampal microglia, along with cognitive impairment. In this sense, Elzinga et al. explore the role of cGAS/STING (highly expressed in microglia) specific signaling in the CNS during obesity and prediabetes. HFD promotes weight gain and dysregulated glucose metabolism in cGAS^−/−^ mice, the onset of which appears to be sexually dimorphic. In particular, HFD induces microglial reactivity primarily in female cGAS^−/−^ mice, but impairs the cognitive function in male, and not female, animals.

In the clinic, the preclinic live animal models used for neurotrauma and neurodegenerative diseases are typically young adults, failing to represent the age of humans. Therefore, this problem in the age difference between human populations and animal models impedes the understanding of the pathological mechanisms of most neurological disorders and the translation of their respective promising therapies. The perspective article by Sefiani analyzes the use of aged primary adult neuronal, microglial, and astrocyte cells during screening phases to significantly increase the translation rate of hits in geriatric patients suffering from neurotraumatic and neurodegenerative diseases.

Astrocytes, the most abundant type of glial cells in the brain, are an integral part of quad partite synapses capable of sensing and modifying synaptic activity. This cell type senses the activity of neighboring neurons and provides feedback by releasing gliotransmitters, including D serine (co-agonist of NMDA receptors). Abreu et al. study astrocytes' contribution to D-serine-mediated NMDAR signaling. In physiological conditions, astrocytic function and gliotransmission (D-serine) play a critical role in maintaining synaptic plasticity and transmission, although it is important to mention that there is a cooperation between neurons and astrocytes in governing D-serine dynamics.

Last but not least, the work by Lee et al. discusses the advantages of using designer receptor exclusive activated by designer drugs (DREADDS) as a powerful *in vivo* technique to study the functional aspects of astrocyte activity and function during cognitive processes, such as memory. The use of this novel approach facilitates the study of G-protein coupled receptor (GPCR)-mediated intracellular calcium (Ca^2+^) and cyclic adenosine monophosphate (cAMP) dynamics, providing access to the intersection between astrocytic GPCR physiology and synaptic function and behavior.

## Author contributions

DC-C: Conceptualization, Writing—original draft, Writing—review & editing. ES-P: Writing—original draft, Writing—review & editing. AO: Writing—original draft, Writing—review & editing.

